# A graph neural architecture search approach for identifying bots in social media

**DOI:** 10.3389/frai.2024.1509179

**Published:** 2024-12-20

**Authors:** Georgios Tzoumanekas, Michail Chatzianastasis, Loukas Ilias, George Kiokes, John Psarras, Dimitris Askounis

**Affiliations:** ^1^Decision Support Systems Laboratory, School of Electrical and Computer Engineering, National Technical University of Athens, Athens, Greece; ^2^DaSciM, LIX, Ecole Polytechnique, Institut Polytechnique de Paris, Palaiseau, France; ^3^Laboratory of Electrical Machines and Installations, Division of Electrical, Electronics and Informatics, School of Engineering, Merchant Marine Academy of Aspropyrgos, Aspropyrgos, Greece

**Keywords:** bot detection, graph neural networks, neural architecture search, propagation, transformation, social media platform X

## Abstract

Social media platforms, including X, Facebook, and Instagram, host millions of daily users, giving rise to bots automated programs disseminating misinformation and ideologies with tangible real-world consequences. While bot detection in platform X has been the area of many deep learning models with adequate results, most approaches neglect the graph structure of social media relationships and often rely on hand-engineered architectures. Our work introduces the implementation of a Neural Architecture Search (NAS) technique, namely Deep and Flexible Graph Neural Architecture Search (DFG-NAS), tailored to Relational Graph Convolutional Neural Networks (RGCNs) in the task of bot detection in platform X. Our model constructs a graph that incorporates both the user relationships and their metadata. Then, DFG-NAS is adapted to automatically search for the optimal configuration of Propagation and Transformation functions in the RGCNs. Our experiments are conducted on the TwiBot-20 dataset, constructing a graph with 229,580 nodes and 227,979 edges. We study the five architectures with the highest performance during the search and achieve an accuracy of 85.7%, surpassing state-of-the-art models. Our approach not only addresses the bot detection challenge but also advocates for the broader implementation of NAS models in neural network design automation.

## 1 Introduction

Social media are online community platforms and apps that let users create, share, and interact with each other's content. Social media content can be text, photos, videos, GIFs, audio, etc. Social media can be used for various reasons, from users who share interests communicating to getting informed about current worldwide events. Social media can also be used for detecting early signs of stress and depression (Ilias and Askounis, 2023b; Ilias et al., 2024b; Kerasiotis et al., 2024). The existence of social media in our day-to-day lives is more prevalent than ever. As of 2023, there are roughly 4.9 billion social media users, a percentage that is more than 60% of the entire population and more than 100 social media platforms. X, previously known as Twitter, stands out as one of the most widely recognized social media platforms. Twitter was launched in 2006. It revolves around the concept of “following” other users. A user can follow accounts they are interested in and see their tweets in their timeline (“following”) and conversely can have “followers” that see their tweets. Nowadays, it has been established as a powerful tool for real-time news updates, public discourse, and social movements, and continues to evolve and enhance its user experience. In 2023, Twitter was renamed to X by then-CEO Elon Musk. The extensive presence of social media in the modern landscape has led to the emergence of accounts that automate interactions on social media platforms, often mimicking human behavior, the so-called bots. These bots can be coded to perform a variety of tasks, such as automatically publishing content, liking, sharing, following, or commenting on posts. Some can even be programmed to engage in conversations to promote specific agendas. Their behavior differs depending on their intent and purpose, but they might share features, such as very high or very low activity levels and more structured and characteristic language patterns (Alsmadi and O'Brien, 2020). Uyheng et al. (2022) examined the origin and traits of trolling messages, finding that they often originate from automated bots and are distinguished by their use of abusive language, reduced cognitive complexity, and specific targeting of individuals or entities. Their study also noted a tendency for bots to target right-leaning sources of information, while trolls tended to engage with less polarized content, spreading misinformation across diverse audiences. Bots are very efficient in spreading misinformation, particularly when programmed with optimized values for factors like walking speed, network distribution, and strategy (Zhang et al., 2024). Fake news and bots have had significant tangible consequences in several cases. Users tend to believe conspiracy theories and misinformation, and correction attempts can sometimes backfire (Xu et al., 2023). Users might also share fake news for altruistic or self-promotional purposes, yet those with greater social media literacy are better equipped to identify and refrain from spreading fake news (Mi and Apuke, 2024). Therefore, there's a need for measures to promote truthful reporting in media and detect any cases of misinformation dissemination.

The need to detect bot accounts to shut them down is quite immediate, assessing the hazards of their uncontrollable presence on social media. Several studies to identify bots from real users have been conducted that provide satisfactory results. There have been several approaches, including supervised learning (Lee et al., 2021), unsupervised learning (Cresci et al., 2017), reinforcement learning (Alauthman et al., 2020), and GNN-based architectures (Feng et al., 2021c). However, all these traditional neural architectures often rely on fixed parameters that are manually designed. Constructing efficient neural network architectures requires extensive feature engineering and can be a quite challenging and time-consuming procedure. Also, fixed architectures often mitigate the models' adaptability on other datasets and tasks. Motivated by these limitations, we examine the implementation of Neural Architecture Search (NAS) to automate the process of discovering optimal architectures. NAS explores a search space of possible architectures and identifies the configurations that enhance the model's performance.

A Neural Architecture Search method that has been proposed to solve the performance issues of fixed architectures is Deep and Flexible Graph Neural Architecture Search (DFG-NAS) (Zhang et al., 2022). It employs an evolutionary algorithm to explore a vast space of permutations of Propagation and Transformation operations, to find the one with the best accuracy in the validation set. Addressing the limitations of previous bot detection models due to their fixed architectures we employ DFG-NAS on a GNN-based approach for bot detection. This approach leverages the user's semantical and property information and constructs a heterogeneous graph out of the follower-following relationships between users. Then, we adapt the DFG-NAS approach to handle Relational Graph Convolutional Neural Networks (RGCNs). The model automatically searches for the permutation of Propagation (P) and Transformation (T) functions, the two main processes of the message-passing protocol, with the highest validation accuracy. The model is also amplified with the use of the Gate operation on the P connections and the use of the skip-connection operation on the T connections.

To the authors' knowledge, DFG-NAS has not been employed before in the task of bot detection. All our experiments were performed on the Twibot-20 dataset (Feng et al., 2021b). The following sums up the contributions of our work:

- We implement DFG-NAS, tailored to RGCNs, to automatically determine the most effective permutation of the message-passing operations.
- We perform experiments to demonstrate the benefits of architecture search in bot detection and compare our method to state-of-the-art models.
- We perform a thorough ablation study on the necessity of the user metadata in our graph, the Gate operation, and the skip-connection operation in NAS.

## 2 Research objective

It is evident to any social media user that bots continue to dominate the digital landscape despite extensive efforts in bot detection and platform initiatives to stop their activities. As technology advances, bots are programmed to mimic human mannerisms more effectively, making them more resilient against detection mechanisms. Beyond the irritation they pose to everyday users, some bots can have tangible and detrimental effects on human society. In 2016 fake news stories spread widely during the U.S. presidential election campaign aiming to influence the public vote (Bessi and Ferrara, 2016). Throughout the COVID-19 pandemic (Ferrara, 2020), bots spread misinformation about the virus and the vaccines on social media, leading to mob panic, confusion, and even resistance to public health measures. Fake news is often framed in a manner that fosters negativity in social discussions and hinders individuals' ability to consider diverse perspectives, contributing to the formation of “echo chambers” on social media platforms (Scheibenzuber et al., 2023). Bots also exacerbate cyberbullying by mass-targeting users, leading to serious psychological consequences. Social media platforms face challenges in effectively moderating such content. Cyberbullying detection methods often rely on unclear definitions and are prone to biases in data annotation (Mahmud et al., 2023). Their evolving nature raises concerns about the efficiency of current preventive measures, highlighting the need for innovation to prevent the dangers posed by this digital phenomenon.

The motivation for this research was constructing a model characterized by adaptability across future datasets, ensuring resilience in the face of evolving technology through time. Many contemporary models rely on fixed architectures, often struggling to demonstrate their efficiency on novel datasets. Although Neural Architecture Search (NAS) has shown significant advantages in various test cases, its application to bot detection remains relatively underexplored, with limited but promising results noted in studies such as Yang et al. (2023). Considering the dynamic nature of the social media landscape and the continuous evolution of bots, more flexible architectures specifically designed for bot identification could offer a practical solution to mitigate their real-world consequences.

This research aims to showcase the efficiency advantages of architecture search and perhaps pave the way for more implementations of NAS models in bot detection in the ongoing battle against automated malicious activities.

## 3 Related work

### 3.1 Bot and fake news detection models

The task of bot identification has attracted numerous studies and many state-of-the-art models propose fascinating methodologies. We could mainly divide these models into supervised learning approaches, unsupervised learning approaches, and GNN-based approaches. In this section, we present some baseline models proposed for bot detection and discuss how they fall into the above categories.

Lee et al. (2021) applied various machine learning algorithms, including SVMs, Naive Bayes, and decision trees, to build and evaluate a supervised bot detection model. The features used in their analysis included account-based features (e.g., the number of followers, friends, tweets), temporal features (e.g., time of account creation, tweet frequency), and content-based features (e.g., usage of URLs, hashtags). Kudugunta and Ferrara (2018) suggested a deep learning model that uses the user's tweets and some metadata features. This architecture includes a tokenizer, GloVE embedding layer, LSTM, and Dense layers. Wei and Nguyen (2019) used only users' tweets with no context of prior knowledge on user profiles, friendship networks, or behavior. They proposed a recurrent neural network (RNN) model that used word embeddings to encode tweets, a three-layer Bidirectional LSTM (BiLSTM), and a softmax layer at the binary output. Cai et al. (2021) proposed their model (BeDM) that involved deep neural networks in bot detection. They employed convolutional neural networks (CNNs) and LSTM, using only the tweet semantics, such as the frequency and the type of publications. Botometer is a web-based program developed by Davis et al. (2016) at Indiana University. It leverages more than 1,000 features to classify Twitter accounts as bots and humans, such as friends, the structure of the social network, user meta-data, temporal activity, and sentiment analysis. Botometer distinguishes the accounts by an overall bot score (ranging from 0 to 5), along with several other scores. The greater the score, the greater the probability that this account is linked to a bot. Yang et al. (2022) presented a thorough introduction of the latest version of Botometer for new users and demonstrated a case study. Alarfaj et al. (2023) utilized features based on content attained from the Twitter API and employed state-of-the-art classifiers, like MLPs, random forest, and naive Bayes. Features included messages, special characters, sentiment analysis, etc. Alothali et al. (2022) introduced their framework, called Bot-MGAT, which stands for bot multi-view graph attention network. The scientists pointed out that other approaches couldn't adjust to different datasets since there wasn't enough recently updated labeled data, which made sense given the constantly shifting behavior of the bots. They presented a methodology that makes use of transfer learning (TL) to leverage the multi-view graph attention mechanism. The framework also benefited from semi-supervised learning, using labeled and unlabeled data. The authors used the TwiBot-20 (Feng et al., 2021b) due to its graph structure, extracting 18 features for the training. Feng et al. (2021a) suggested SATAR. In particular, SATAR leverages the user's semantics, property, and neighborhood information. It adjusts by fine-tuning parameters and pre-training on a huge number of self-supervised users. The authors proposed two alternative models: *SATAR*_*FC*_ and *SATAR*_*FT*_. Ilias and Roussaki (2021) proposed two methods for bot detection using deep learning techniques. Their first approach extracts a substantial 71 features per user to utilize for account classification to bots and genuine users. They also employed various feature selection techniques to discard redundant and irrelevant features. Their second methodology proposes a deep learning architecture for tweet-level classification. This architecture incorporates an attention mechanism atop the Bidirectional Long Short-Term Memory (BiLSTM) layer. During the learning phase, the attention mechanism helps the model better focus on relevant information. Ilias et al. (2024a) focused solely on user descriptions and sequences of actions performed by Twitter accounts. Their approach includes both unimodal (text or image) and multimodal (both text and image) methods. They designed digital DNA sequences per user based on tweet type and content, converted these sequences into 3D images, and fine-tuned pre-trained vision models like AlexNet, ResNet, and VGG16. For bot detection through user descriptions, they fine-tuned TwHIN-BERT, a transformer model. In multimodal approaches, they use VGG16 for visual representation and TwHIN-BERT for textual representation, proposing three fusion methods: concatenation, gated multimodal unit (GMU), and cross-attention. They conducted their experiments on both the Cresci'17 and TwiBot-20 dataset. Wei and Nguyen (2023) proposed their model BOTLE. Their model utilizes a recurrent neural network (RNN) with Bidirectional Gated Recurrent Units (BiLGRU) connecting two hidden layers of opposite directions leading to the same output. Notably, BOTLE does not rely on handcrafted features or pre-existing information regarding account profiles. Linguistic embeddings, including word, character, part-of-speech, and named-entity embeddings, are employed to encode tweet content, eliminating the need for labor-intensive feature engineering. Bazmi et al. (2023) introduced the Multi-View Co-Attention Network (MVCAN), which aims to capture the latent topic-specific credibility of both users and news sources. This model represents news articles, users, and news sources in a manner that encodes topical viewpoints, socio-cognitive biases, and partisan biases as vectors. These features are encoded using a variant of the Multi-Head Co-Attention (MHCA) mechanism. Shevtsov et al. (2023) introduced their model BotArtist, constructed on a semi-automatic machine learning pipeline, that requires minimal features for training, taking into consideration the loads of data needed by previous approaches and the recent monetization of Twitter API requests. Sujith et al. (2022) proposed a supervised learning approach that used multiple models to detect bots. Their classification of accounts relied on features like user metadata, tweet content, and posting history, among others. In addition to identifying bot accounts, the authors assigned a level of significance or influence to them, prioritizing the removal of the most influential or harmful bot accounts. Liu et al. (2023) proposed BotMoE, which leverages three perspectives of user information (metadata, text, and graph representations) and incorporates a community-aware Mixture-of-Experts (MoE) layer to assign users to different communities. The user representations are fused with an extractor fusion layer and supervised learning is employed to train the BotMoE framework to perform community-aware bot detection. Saxena et al. (2023) proposed two frameworks for recognizing accounts that disseminate false information on Twitter. Initially, they employed profile-based data, including the verified status, profile photo, and account lifetime and activity. Then, they combined tweet-propagation patterns and assigned a credibility score to each user, signifying their authenticity. Dimitriadis et al. (2024) proposed CALEB that is based on the Conditional Generative Adversarial Network (CGAN) and its extension, Auxiliary Classifier GAN (AC-GAN). By developing realistic artificial bot varieties, they were able to replicate the evolution of bots. As a result, they enhanced already-existing datasets and were able to identify bots before they emerged.

Yang et al. (2020) used a combination of unsupervised and supervised learning methods for bot detection. Specifically, the authors utilized minimal features derived from user metadata, temporal patterns, network structure, sentiment analysis, and linguistic cues that they fed into a machine learning pipeline, that reduced dimensionality and included classification algorithms. Cresci et al. (2017) introduced the Social Fingerprinting technique for bot detection, a Digital DNA technique that models social network users' behaviors. Each user is represented as a sequence of characters depending on the type and content of the tweets they publish, simulating a DNA sequence. The authors try to find similarities in the sequences defining the length of the Longest Common Substring (LCS) between two sequences. For a set of real users, the length of LCS was found to be particularly small, leading to the conclusion that longer sequences than the average LCS were bots. Based on this idea, the authors developed two techniques, one based on supervised learning and another on unsupervised learning to find similarities in the behavior of accounts. Quezada et al. (2023) developed a real-time bot infection detection model that analyzes Domain Name System (DNS) traffic events. They extracted 13 attributes from DNS logs to create unique fingerprints for servers. Using Isolation Forest, an algorithm for unsupervised learning, they identified anomalies in the fingerprints to classify hosts as infected or not. The model also utilized Domain Generation Algorithms (DGA) to search for queries to anomalous domains. Finally, a Random Forest, a supervised learning algorithm, was employed to create a model for detecting future bot infections on hosts. Miller et al. (2014) approached bot identification as an anomaly detection problem. They extracted 107 features from user's tweets and property information and adapted two stream clustering algorithms, StreamKM++ and DenStream, to facilitate spam detection and identified bot users as abnormal outliers. Chavoshi et al. (2016) developed DeBot, a bot detection system for social media, using warped correlation to identify likely bot accounts based on their high synchronicity, a characteristic unlikely in human users. DeBot doesn't require labeled data and operates on activity correlation. Moreover, through the utilization of a lag-sensitive hashing technique, it can promptly cluster accounts for real-time classification. Minnich et al. (2017) proposed their real-time unsupervised model BotWalk. Using metadata, content, temporal, and network features they employ anomaly detection, comparing each user to a seed bank of labeled accounts iteratively. Mannocci et al. (2022) proposed MulBot, an unsupervised bot detection system that utilizes multivariate time series (MTS) analysis. They employed an LSTM autoencoder to map the MTS data into a latent space and then conducted clustering on this encoded data to find dense clusters of users exhibiting similar behavior, assuming this was a common trait of bot accounts. MulBot also showcases effectiveness in identifying and distinguishing various botnets. Wu et al. (2022) employed unsupervised machine learning techniques, specifically K-Means and Agglomerative clustering, for Twitter bot detection. They used account activity, popularity, and verification status, among other features for the clustering. Koggalahewa et al. (2022) introduced an unsupervised method for bot identification based on a user's peer approval in the social network. They based peer acceptance between two users on their shared interests over a multitude of issues. Lopes et al. (2022) introduced their botnet identification model, designed to detect networks of compromised devices under master control. Their approach relies on analyzing network flow behavior through a contemporary method known as the Energy-based Flow Classifier (EFC). EFC employs inverse statistics to enhance anomaly detection.

Ali Alhosseini et al. (2019) introduced the use of graph convolutional neural networks (GCNN) in bot identification. They noted that besides the users' features, the construction of a social network would enhance a model's ability to distinguish the bots from the genuine users. Feng et al. (2022) introduced the aspect of diversity in relationships and influence dynamics among users in the Twittersphere for bot detection. They proposed a bot detection framework that leverages a network with users as nodes and the different relations as edges. Then they aggregated messages across users and operated heterogeneity-aware Twitter bot detection. They conducted their experiments using the Twi-Bot20 dataset. Feng et al. (2021c) proposed their model for bot detection BotRGCN, which is short for Bot detection with Relational Graph Convolutional Networks. BotRGCN builds a heterogeneous graph out of the following relationships and uses information, such as the user's description, tweets, numerical and categorical property set, and neighborhood information. The experiments were conducted on the Twi-Bot20 dataset (Feng et al., 2021b), but BotRGCN could exploit other types of relations if supported by the dataset. Kušen and Strembeck (2020) examined the structural dynamics of conversations between humans and bots on Twitter following emotionally charged riot events. They introduced “emotion-exchange motifs” to identify recurring patterns in emotional message exchanges. Their findings revealed that human conversations exhibited various motifs with reciprocal edges and self-loops, indicating interactive dialogue. In contrast, bots typically disseminated identical messages to multiple users or did not anticipate replies. Moreover, bots frequently initiated conversations and often conveyed fear-inducing messages. Bui and Potika (2022) introduced a graph-based method for bot detection. They detailed their data collection process and identified specific behaviors indicative of an account being associated with a bot. These behaviors can include engagement with other users, nonsensical usernames and profile information, repetitive content posting, and retweeting activity. These observations are utilized to label the accounts accordingly. Dehghan et al. (2023) suggested that the local social network formed around each account can aid in identifying the bots. To prove their hypothesis, they compared two classes of embedding algorithms, the former of which focused on proximity data and the latter that focused on nodes' neighborhoods. They discovered that the structural embeddings presented higher information underlining the valuable information that is embedded within each node's local network. Pham et al. (2022) introduced their approach Bot2Vec, which eliminated the need for user profile features. To improve the model's generalization on many social media platforms, they used only local neighborhood relations and the community structure of the graph that represented the users and employed an random walk strategy in the communities. Noekhah et al. (2020) proposed their model “Multi-iterative Graph-based opinion Spam Detection” (MGSD) that aims to identify various types of spam entities. It analyzes all kinds of relationships between them and utilizes domain-independent features, allowing for generalization across types of opinionated documents. Trained on both existing and novel features, MGSD assigns a spam score to each entity. Ye et al. (2023) proposed HOFA, a graph-based framework for bot detection, featuring two key modules: Homophily-Oriented Graph Augmentation (Homo-Aug) and Frequency Adaptive Attention (FaAt). The Homo-Aug employs an MLP to extract user representations and generate a k-NN graph. Meanwhile, the FaAt module acts as a low-pass filter for homophilic edges and a high-pass filter for heterophilic edges. This function prevents excessive smoothing of user features by the neighborhood. El-Mawass et al. (2020) explored using the output of existing supervised classification systems to detect spammers. They incorporated the classifiers' outputs as prior beliefs within a probabilistic graphical model framework. Proposing a bipartite users-content interaction graph, they facilitated the spread of beliefs to similar accounts. Constructing a Markov Random Field on a graph of similar users, they employed Loopy Belief Propagation to derive the predictions. Their findings demonstrated a notable enhancement in recall while maintaining precision.

### 3.2 Neural architecture search approaches

Neural architecture search can increase performance in many tasks (Chatzianastasis et al., 2023). Graph neural architecture search is proposed as the solution to performance limitations due to a fixed architecture. Parameter tuning in neural networks can be a challenging task. Many NAS methods have been suggested that include variations in the search space, the optimization method, and the architecture evaluation. We will divide these methods based on their optimization method, which will include reinforcement learning, evolutionary algorithms and gradient-based methods.

Zhou et al. (2022) proposed the automated graph neural networks (Auto-GNN) framework. Auto-GNN searches for the best GNN architecture possible in a predetermined search space, divided into six classes of actions: hidden dimension, attention function, attention head, aggregate function, combine function, and activation function. For efficiency reasons, the authors designed a conservative explorer to preserve the optimal neural architecture discovered during the search. The authors also implemented constrained parameter sharing, adapted to the heterogeneous GNN architecture. Two experimental methods were presented: inductive, in which the graph structure and node features on the testing and validation sets are unknown during training, and transductive, which involves the availability of unlabeled data for testing and validation during training. Gao et al. (2021) proposed GraphNAS to implement an automatic search of the best graph neural architecture based on reinforcement learning. The search space covers sampling functions, aggregation functions, and gated functions. GraphNas also uses more efficient parameter-sharing techniques than other contiguous models for CNNs and RNNs. After training 1,000 different architectures, the five best ones were used for the testing, which surpassed human-invented ones or those produced by random searches. Zhao et al. (2020) proposed the SNAG framework (Simplified Neural Architecture Search for Graph neural networks). The suggested framework had two key components: Node aggregators, which focused on neighborhood features, and Layer aggregators, which focused on the range of the neighborhood used. The search space algorithm was a variant of Reinforcement Learning that adopted the weight-sharing mechanism (SNAGWS). Nunes and Pappa (2020) presented one NAS methods for optimizing GNNs based on reinforcement learning and one based on evolutionary algorithms. The authors defined two cases of search spaces: Macro, where the architectures generated have the same structure, and Micro, where the architectures are not rigidly structured but combine several convolutional schemas. They concluded that EA and RL found very similar architectures to those found by a random search, a significantly simpler technique. However, they pointed out that whilst the other approaches generated large structures in as much as 80% of the situations, EA created the majority of GPU-fitting designs. Li et al. (2023) proposed Meta-GNAS that uses meta-reinforcement learning from past tasks to apply that knowledge to new tasks. Additionally, they speed up the search by using a predictive model to evaluate the potential graph neural architectures instead of training them from scratch.

Peng et al. (2020) implemented a NAS approach to human action recognition from skeleton movements. The search space was enlarged with diverse spatial-temporal graph modules while constructing higher-order connections between nodes using Chebyshev polynomial approximation. The search algorithm used is an evolutionary adaptation with a high sampling efficiency, denoted Cross-Entropy method with ImportanceMixing (CEIM). Jiang and Balaprakash (2020) adapted the method of neural architecture search to the conception of GNNs for predicting molecular properties. The authors designed neural networks for message-passing (MPNNs) between nodes. To find an optimal MPNN from the user-defined search space, they used regularized evolution (RE) from the DeepHyper package. Zhang et al. (2022) proposed DFG-NAS, an innovative method that allows for automatic search of very deep and adaptable GNN architectures. DFG-NAS focuses on exploring macro-architectures, specifically the implementation details of atomic propagation (P) and transformation (T) operations within the GNN. P is linked to the graph structure, whereas T concentrates on the non-linear transformations within the neural network. In addition, they adopted gating and skip-connection mechanisms for deeper GNN pipelines. They used an evolutionary algorithm to find the optimal architecture, which supported four cases of mutation. Peng et al. (2023) introduced Fast-ENAS as a computationally efficient alternative to Evolutionary Neural Architecture Search. This method utilizes a training-free performance metric that is computed with a single forward pass. The authors enhance the search process by incorporating a GCN-based contrastive predictor, aiming to improve the accuracy of the estimated performance of a candidate architecture, bringing it closer to its actual performance. Shang et al. (2023) introduced AG-ENAS, which brings two key innovations to the Evolutionary Neural Architecture Search process. Firstly, it employs an adaptive parameter adjustment mechanism based on population diversity and fitness, enhancing the adaptation of genetic operators' associated parameters. Secondly, the model introduces a mutation operator guided by the gene potential contribution. It improves offspring quality by assigning weight to more valuable genes through a distribution index matrix. The concept of aging is integrated into environmental selection to mitigate premature convergence. Lopes et al. (2024) presented Guided Evolutionary Architecture (GEA), which tackles the problem of other NAS models getting trapped in suboptimal solutions during the search process. GEA overcomes this challenge by generating and evaluating multiple architectures using a zero-proxy estimator and selecting only one with the best-performing one for the next generation. This approach expands the search space without increasing complexity, as new architectures are derived from previous ones through mutations.

Zhao H. et al. (2021) proposed their framework SANE. The search space has similarities with the search space from the SNAG framework, with Node and Layer aggregators. However, the authors presented a novel differentiable search algorithm. Cai et al. (2021) introduced a GNAS approach featuring a uniquely designed search space and a gradient-based search approach. The authors developed a three-level Graph Neural Architecture Paradigm (GAP) that includes two types of fine-grained atomic operations (neighbor aggregation and feature filtering) that are derived from message-passing, to build the search space. Li et al. (2021) introduced an innovative dynamic one-shot search space designed for multi-branch neural architectures within GNNs. The dynamic nature of the search space offers a larger capacity than a larger predefined search space. The architectures with lower importance weights are removed periodically from the population, while the candidate operations are unique to every edge of the computational graph. The authors performed both supervised and unsupervised techniques for the training part. Zhao J. et al. (2021) proposed a gradient-based architecture search method for predicting a system's remaining useful life. Their approach models the search space as a directed acyclic graph (DAG), where nodes represent latent representations and edges represent transformation operations. By employing candidate operations like ReLU and tanh, along with the softmax function, they make the search space continuous and the objective function differentiable, facilitating gradient-based optimization methods to find the optimal architecture.

### 3.3 Related work review findings

From the aforementioned research works, it is clear that there have been many approaches to the task of bot detection. Previous studies include supervised, unsupervised, and graph neural network (GNN) based methods. While they have shown promising results, the relentless evolution of bot accounts toward simulating human-like patterns poses a significant challenge to their effectiveness. These models are constrained by fixed architectures, limiting their adaptability to newer datasets.

Little work has been done in employing Neural Architecture Search methods in GNN-based methodologies for bot detection. Our work shifts the focus on overcoming the performance limitations due to fixed architectures, by utilizing DFG-NAS to search for the best configuration of Propagation and Transformation functions in the message passing protocol of our RGCNs. Instead of extensive feature engineering our model searches for the permutation with the highest accuracy and aims for better adaptability in newer datasets that will depict future bots' behavior. Moreover, DFG-NAS presents high advantages, as it is suitable for GNN-based methods and overcomes over-smoothing and model degradation issues with the gate and skip-connection operations.

## 4 Dataset

The TwiBot-20 Dataset (Feng et al., 2021b) is a publicly available dataset, constructed with a breadth-first search (BFS) methodology. The dataset includes information about each user's profile information obtained from the Twitter API, recent tweets, and domains of the user's interest. It also contains information about the user's neighborhood, which helps us construct a heterogeneous graph from the following relationships. Table 1 presents all the attributes of the TwiBot-20 Dataset and a short description of them. The information from the user profiles is further mentioned in the preprocessing part of the model. The graph that is constructed consists of 229,580 nodes and 227,979 edges. The objective of the bot detection system is to distinguish between bots and genuine users by analyzing information from user descriptions, tweets, numerical and categorical properties, as well as neighborhood information.

**Table 1:** TwiBot-20 dataset attributes.

**Attribute**	**Description**
ID	ID from Twitter to identify the user
Profile	Profile information from Twitter API
Tweet	200 recent tweets of the user
Neighbor	20 random followers and followings of the user
Domain	Domain of the user (politics, business, entertainment, sports)
Label	Label of the user (“1”: bot, “0”:human)

## 5 Methodology

In this part, we present a complete analysis of our methodology. First, we describe the preprocessing of the user metadata used in our model. Next, we introduce the use of Relational Graph Convolutional Neural Networks and the two functions in Message Passing. Last, we explain the use of DFG-NAS (Zhang et al., 2022) in searching for the best permutation of Propagation and Transformation functions. In Figure 1, we depict the architecture of the model on a higher level, while Figure 2 presents the connections between the different layers of an example configuration of P and T functions.

**Figure 1A:**
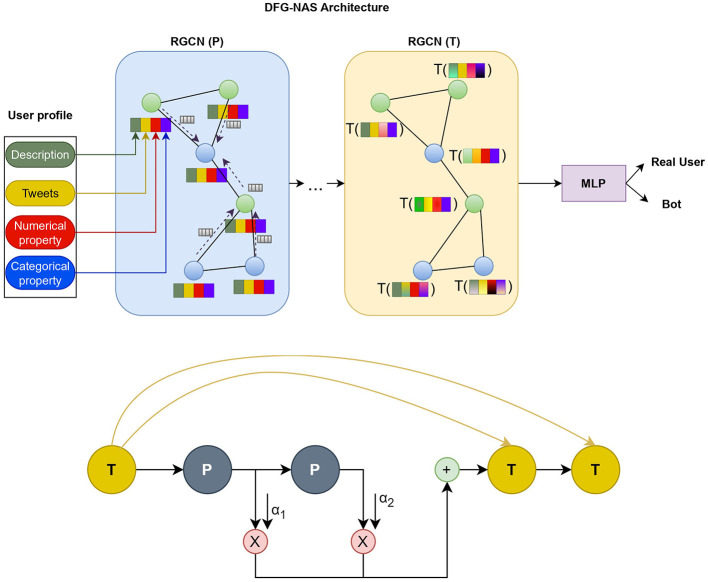
Model used for Bot detection. User metadata is fed to the architecture proposed by NAS. The P step includes message aggregation from neighbor nodes. The T step includes the transformation process on each node based on neighbor relations. In the final part, an MLP decides whether the account belongs to a real user or a bot.

**Figure 1B:**
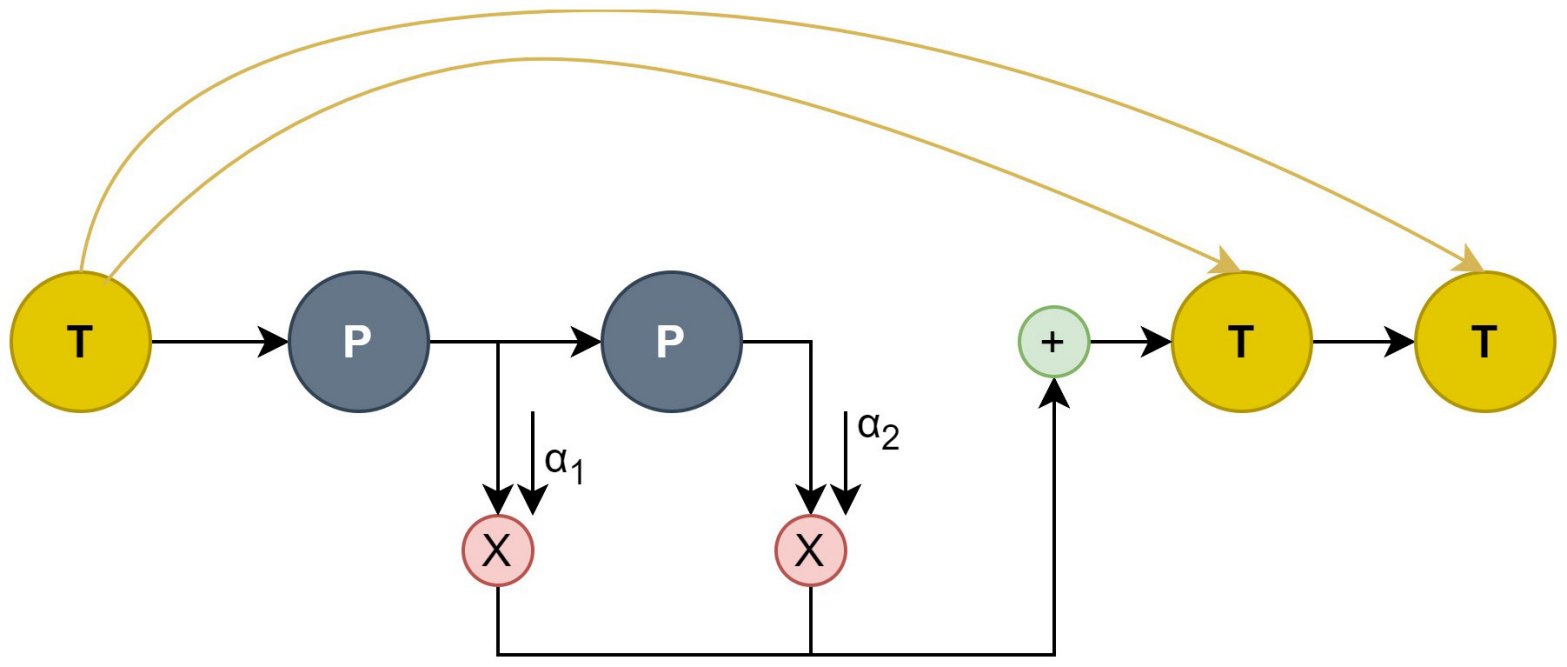
Example of connections between the layers of NAS architecture. New T steps congregate information from all previous T steps. P steps propagate their embeddings and sum them up for the next T step.

**Figure 2:**
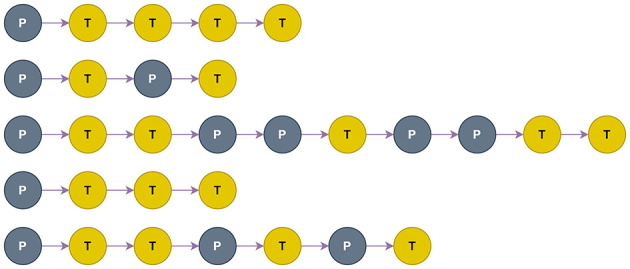
Permutations of Propagation (P) and Transformation (T) functions of the top-5 performing architectures from DFG-NAS. Their validation accuracies in the architecture search (from up to down) are: 87.01%, 86.99%, 86.95%, 86.89%, 86.82%.

### 5.1 Data preprocessing

We follow the preprocessing suggested by Feng et al. (2021c) for BotRGCN. Each user's representation includes metadata that are preprocessed as follows:

- Overall: user's description, tweets, numerical and categorical properties are encoded and concatenated to finally represent the user's metadata:

(1) $$\begin{array}{l} r=\left[r_{b};r_{t};r_{p}^{num};r_{p}^{cat}\right]\in {\mathbb{R}}^{D\times 1} \end{array}$$

where *D* is the user embedding dimension. Each feature's procession and representation are explained below. Later we will prove that the model's performance is attributed to all these features and not only to the heterogeneous graph.

- User description: the user descriptions are encoded with pre-trained RoBERTa:

(2) $$\begin{array}{l} \overset{-}{b}=RoBERTa\left({\left\{b_{i}\right\}}_{i=1}^{L}\right),\bar{b}\in {\mathbb{R}}^{D_{s}\times 1} \end{array}$$

where $\bar{b}$ denotes the user description representation and *D*_*s*_ is the dimension of the RoBERTa embedding. The vectors for the user's description are derived:


(3) $$\begin{array}{l} r_{b}=\phi \left(W_{B}\cdot \bar{b}+b_{B}\right),r_{b}\in {\mathbb{R}}^{D/4\times 1} \end{array}$$

where *W*_*B*_ and *b*_*B*_ represent trainable parameters, ϕ denotes the activation function, and *D* is the dimension of the embedding.

- User tweets: the user tweets are also encoded using RoBERTa. The ultimate representation of the user's tweets, denoted as *r*_*t*_, is computed as the average of the representations of all individual tweets.
- User numerical properties: the user's numerical properties are adopted straight from the Twitter API with no feature engineering and presented in Table 2. For this information *z*-score normalization is conducted to get the representation $r_{p}^{num}$ from a fully connected layer.
- User categorical properties: the user's categorical properties are also encoded with MLPs and GNNs, without feature engineering, just as the numerical properties. They are adopted straight from the Twitter API and presented in Table 3. After one-hot encoding, they are concatenated and transformed through a fully connected layer and leaky-relu to get their representation $r_{p}^{cat}$.

**Table 2:** User numerical properties.

**Feature name**	**Description**
#followers	Number of followers
#followings	Number of followings
#favorites	Number of likes
#statuses	Number of statuses
active_days	Number of active days
screen_name_length	Screen name character count

**Table 3:** User categorical properties.

**Feature name**	**Description**
protected	Protected or not
geo_enabled	Geo-location enabled or not
verified	Verified or not
contributors_enabled	Enable contributors or not
is_translator	Is translator or not
is_translation_enabled	Translation or not
profile_background_tile	The background tile
profile_user_background_image	Background image or not
has_extended_profile	Extended profile or not
default_profile	The default profile
default_profile_image	The default profile image

### 5.2 Relational graph convolutional neural networks

Our method builds a heterogeneous graph out of the following relationships. Users are considered nodes and the “following” and “followers” relations are represented as edges connecting the nodes. The user's “followers” are therefore represented differently than the user's “following.” The heterogeneous graph that is constructed can represent better the relations between users and more relations between the users could be integrated into the graph if supported by the dataset. The users also contain the concatenated metadata that we described below.

To combine the users' representations with the relationships between users we make use of RGCNs. The message-passing process in RGCNs comprises two fundamental operations: propagation (P) of the representations of the user's neighbors and transformation (T) on these representations. Below we describe the process behind the two functions:

- Propagation (P): propagation includes message aggregation from neighbor nodes without explicit node feature transformation. The mathematical expression for the propagation step is as follows:
  (4) $$\begin{array}{l} h_{i}^{\left(l+1\right)}=\underset{r\in R}{\sum }\underset{j\in N_{i}^{r}}{\sum }\frac{1}{c_{i,r}}W_{r}^{\left(l\right)}h_{j}^{\left(l\right)} \end{array}$$
  where $h_{i}^{\left(l+1\right)}$ is the new node feature after propagation, *R* is the set of relations, $N_{i}^{r}$ are the neighbors of the node with relation *r*, *c*_*i,r*_ is a normalization constant that can be learned or chosen in advance (for example *c*_*i,r*_ = $N_{i}^{r}$) and $W_{r}^{\left(l\right)}$ is the learnable weight matrix for relation *r*.
- Transformation (T): transformation occurs on each node based on the relations. The mathematical expression for the transformation step is as follows:
  (5) $$\begin{array}{l} h_{i}^{\left(l+1\right)}=W_{root}h_{i}^{\left(l\right)}+\underset{r\in R}{\sum }\left(W_{r}h_{i}^{\left(l\right)}\right) \end{array}$$
  where $h_{i}^{\left(l+1\right)}$ is the new node feature after transformation, *W*_*root*_ is the learnable weight matrix for the root node, *W*_*r*_ is the learnable weight matrix for relation r and R is the set of relations.

We segregate these two types of functions since combinations of them will construct the search space for the architecture search.

### 5.3 Graph neural architecture search

The use of Graph Neural Networks offers undeniable advantages in the task of bot detection. However, maximizing their performance may require extensive feature engineering. This is why we employ Graph Neural Architecture Search, using the model DFG-NAS (Zhang et al., 2022). Thus, we search for the permutation of Propagation and Transformation steps that achieves the highest accuracy. Most G-NAS methods have a fixed pipeline length since the performance decreases with too many P operations as the layers become deeper, which is referred to as the over-smoothing issue. Propagation and transformation operations regulate the effect of smoothing. Moreover, with unlimited pipeline length DFG-NAS searches for more flexible pipelines of P and T operations, using an evolutionary algorithm. It also makes use of gating and skip-connection mechanisms in the P and T operations, respectively.

The search space includes P-T combinations and the number of P-T operations. The output of node *v* in the l-th layer is represented by $o_{v}^{\left(l\right)}$ in a single P or T operation within a single GNN layer of the model. The layer indices of all P and T operations are included in two sets, *L*_*P*_ and *L*_*T*_. The connections of P and T are depicted in Figure 1B and also described below.

#### 5.3.1 Propagation connections

An imminent problem in GNNs is over-smoothing or under-smoothing, a problem that arises with too many or too few propagation operations. To achieve suitable smoothness for different nodes, the P operations are amplified with a gating mechanism. If the next operation is also P, the result of the l-th P operation is the propagated node embedding of *o*^(*l*−1)^. On the other hand, if T is the next operation, a node-adaptive combination weight is allocated for the node embeddings propagated by all of the previous P operations. Formulatively:


(6) $$\begin{array}{l} z_{v}^{\left(l\right)}=P\left(o_{v}^{\left(l-1\right)}\right) \end{array}$$

(7) $$o_{v}^{\left(l\right)}=\{\begin{array}{ll} z_{v}^{\left(l\right)}, & followed\;by\;P\\ \sum_{i\in L_{P},i\leq l}softmax(a_{i})z_{v}^{\left(i\right)}, & followed\;by\;T \end{array}$$

where $a_{i}=\sigma \left(s\cdot o_{v}^{i}\right)$ represents the weight for the i-th layer output of node *v*. Here, *s* is the learnable vector shared among the entirety of nodes, and σ denotes the Sigmoid function. To ensure proper scaling, the Softmax function is employed to normalize the sum of gating scores, making it equal to 1.

#### 5.3.2 Transformation connections

An imminent issue with GNNs is the model degradation issue, caused by a hyperbolic amount of transformation operations and may result in a reduction of the model's accuracy. To mitigate this issue, skip-connection mechanisms are used in T operations. Each T operation's input is the total of all the T operations' outputs up to the last layer and the output from the layer before it. The input and output of the l-th T operation can be formulated as:


(8) $$\begin{array}{l} z_{v}^{\left(l\right)}=o_{v}^{\left(l-1\right)}+\underset{i\in L_{T},i<m\left(l\right)}{\sum }o_{v}^{\left(i\right)} \end{array}$$

(9) $$\begin{array}{l} o_{v}^{\left(l\right)}=\sigma \left(z_{v}^{\left(l\right)}w^{\left(l\right)}\right) \end{array}$$

where *m*(*l*) represents the index of the last T operation before the l-th layer, and *W*(*l*) denotes the trainable parameter in the l-th T operation.

Evolutionary algorithms are a class of optimization algorithms inspired by biological evolution that aim to achieve the best accuracy in offspring through mutations. In our case, each GNN architecture is represented as a sequence of P and T operations. Each pipeline can be considered a chromosome and the mutations that occur simulate nature's mutations. These mutations can happen at any random position in the sequence. In our instance, four different cases of mutation can be enforced:

- +P: append a propagation operation.
- +T: append a transformation operation.
- P → T: replace a propagation operation with a transformation one.
- T → P: replace a transformation operation with a propagation one.

Initially, *k* distinct GNN designs are generated at random and evaluated on the validation set. These architectures represent the initial population set Q. Subsequently, m (m < k) members of the population are randomly sampled, and parent A is determined by selecting the member with the highest validation accuracy. By enforcing a random mutation of the four presented on A, a child architecture B is produced. B is then evaluated and added to the population, and the oldest person is eliminated. After T generations of this procedure, the architecture with the best performance is eventually returned.

DFG-NAS returns a sequence of P and T steps. As illustrated in Figure 1A, each step consists of an RGCN that conducts one of the two main functions as we described incorporating both the user metadata and the user relations. After the RGCNs layers an MLP is employed to finally distinguish bots from genuine users.

## 6 Experiments

### 6.1 Baselines

We compare our proposed apporach to the state-of-the-art models that are referenced in the paper of BotRGCN (Feng et al., 2021c). These experiments are all ran on the same dataset as the one we used for a fair comparison. We are using the published results for the comparison. More specifically, we compare our model to these state-of-the-art models:

- Lee et al. (2021) employed different supervised algorithms with several user features.
- Yang et al. (2020) used a combination of supervised and unsupervised learning with minimal user features.
- Kudugunta and Ferrara (2018) used both the tweets and the account metadata.
- Wei and Nguyen (2019) employed an RNN model utilizing only the user's tweets.
- Miller et al. (2014) extracted 107 features and employed stream clustering algorithms.
- Cresci et al. (2017) identified bots by computing the longest common substring between encoded sequences of users.
- Botometer (Davis et al., 2016) is a web-based program that leverages more than 1,000 user features.
- Ali Alhosseini et al. (2019) introduced graph convolutional neural networks in bot detection.
- SATAR (Feng et al., 2021a) leverages the user's semantics, property, and neighborhood information
- Feng et al. (2021c) used the user's description, tweets, numerical and categorical properties, and neighborhood information.
- Ilias et al. (2024a) designed two cross-attention layers based on the digital DNA sequence.

### 6.2 Experiment settings

The experiment was run on Google Colab using Nvidia's T4 GPUs. The population set k for the architectural search is 15, and the maximum generation time T is 80. The training budget of each GNN architecture is 70 epochs. These numbers although limited due to our resources, provide a great example of the efficiency of our model. More complex architectures that we tested do not necessarily provide better results. Also, the number of epochs is sufficient to get a good idea of each architecture's accuracy. Adam optimizer is used for training, and its learning rate is set to 0.04. The criterion is Cross Entropy Loss and the regularization factor is 2e-4. Dropout is applied to all feature vectors at a rate of 0.5, and dropout among GNN layers is set to 0.8.

After running the NAS method we process the results and examine the five architectures with the best accuracy in the validation set. Each architecture is now trained with 100 epochs on the TwiBot-20 dataset (Feng et al., 2021b). The train set is 70% of the dataset, the validation set is 20% and the test set is 10%. Adam optimizer with a learning rate of 1e-3 is also used for training. Then each architecture is tested on the test set. We will present the findings of these experiments below.

### 6.3 Evaluation metrics

We assess our model's performance using its Accuracy, F1-score, Precision, Recall, Specificity, and MCC. These metrics are computed by labeling the bots as the positive class and the genuine users as the negative class. To compare the performance of our model to the other baseline models we will only use the metrics Accuracy, F1-score, and MCC.

## 7 Results

Each architecture during the search is saved with its P-T configuration, accuracy in the validation set, and accuracy in the test set. In Figure 2, the five architectures with the highest validation accuracy that are chosen from the NAS method are depicted.

These architectures are trained and tested from scratch in TwiBot-20 dataset. We present all the metrics attained by all the architectures in Table 4.

**Table 4:** Performance of the architectures from architecture search.

**Model**	**Accuracy**	**F1-score**	**Precision**	**Recall**	**Specificity**	**MCC**
1st Architecture	0.852 ± 0.005	0.865 ± 0.008	0.851 ± 0.015	0.880 ± 0.031	0.818 ± 0.027	0.702 ± 0.010
2nd Architecture	0.855 ± 0.004	0.869 ± 0.005	0.853 ± 0.007	0.886 ± 0.012	0.819 ± 0.012	0.709 ± 0.009
3rd Architecture	**0.857** **±0.004**	**0.871** **±0.003**	0.849 ± 0.008	**0.895** **±0.007**	0.812 ± 0.013	**0.712** **±0.007**
4th Architecture	0.852 ± 0.006	0.864 ± 0.008	0.856 ± 0.009	0.873 ± 0.026	0.828 ± 0.018	0.702 ± 0.013
5th Architecture	0.852 ± 0.007	0.864 ± 0.008	**0.858** **±0.003**	0.872 ± 0.019	**0.829** **±0.007**	0.703 ± 0.014

Values are reported as mean ± standard deviation. Five runs of results are averaged. The best outcomes for each evaluation metric are in bold.

All selections achieve good metrics and present advantages in bot detection over state-of-the-art methods. These results underscore the significant advantages that emerge from employing architecture search techniques regarding the field of bot recognition. Moreover, they establish the efficiency of utilizing user features and relationships between users in bot detection.

Upon closer examination of the results, the third architecture achieves the best evaluation metrics. The fifth architecture has the highest precision. However, all the architectures present high metrics of accuracy, F1-score, and MCC and whichever architecture we choose could compete with state-of-the-art models. From now on we will refer to the third architecture as our model, since it provides the highest accuracy.

In Table 5 we present the performance of the baseline methods on the TwiBot-20 dataset compared to ours. We see that our model benefits from the search for the fittest architecture that we performed beforehand, as it achieves a higher accuracy, F1-score, and MCC than other state-of-the-art methods.

**Table 5:** Performance of models on the TwiBot-20 dataset.

**Model**	**Accuracy**	**F1-score**	**MCC**
Lee et al. (2021)	0.7456	0.7823	0.4879
Yang et al. (2020)	0.8191	0.8546	0.6643
Kudugunta and Ferrara (2018)	0.8174	0.7517	0.6710
Wei and Nguyen (2019)	0.7126	0.7533	0.4193
Miller et al. (2014)	0.4801	0.6266	-0.1372
Cresci et al. (2017)	0.4793	0.1072	0.0839
Davis et al. (2016)	0.5584	0.4892	0.1558
Ali Alhosseini et al. (2019)	0.6813	0.7318	0.3543
Feng et al. (2021a)	0.8412	0.8642	0.6863
Feng et al. (2021c)	0.8462	0.8707	0.7021
Ilias et al. (2024a)	0.7466	0.7630	–
**Ours**	**0.8568** **±0.004**	**0.8712** **±0.003**	**0.7116** **±0.007**

Values are reported as mean ± standard deviation. Five runs of results are averaged. The best outcomes for each evaluation metric are in bold.

## 8 Ablation study

To demonstrate our model's effectiveness and integrity we will perform an ablation study on the basic ideas: the user's features used for the training, the Gate operation, and the skip-connection operation.

To prove that using multi-modal information is vital to our model performance we will train the architecture that produces the best results with reduced features. We will reduce one feature at a time and use combinations of the features for the training. We present the results in Table 6.

**Table 6:** Training model with less features.

**Model**	**Accuracy**	**F1-score**	**Precision**	**Recall**	**Specificity**	**MCC**
Ours	0.857 ± 0.004	0.871 ± 0.003	0.849 ± 0.008	0.895 ± 0.007	0.812 ± 0.013	0.712 ± 0.007
w/o description	**0.859** **±0.004**	**0.875** **±0.004**	0.845 ± 0.002	0.906 ± 0.008	0.804 ± 0.004	**0.718** **±0.008**
w/o tweets	0.833 ± 0.007	0.858 ± 0.007	0.796 ± 0.004	0.93 ± 0.013	0.719 ± 0.005	0.671 ± 0.016
w/o numerical	**0.859** **±0.003**	0.872 ± 0.005	**0.856** **±0.012**	0.889 ± 0.023	**0.823** **±0.021**	0.716 ± 0.007
w/o categorical	0.792 ± 0.003	0.814 ± 0.001	0.791 ± 0.010	0.840 ± 0.014	0.738 ± 0.021	0.582 ± 0.005
Des + tweets	0.759 ± 0.007	0.773 ± 0.009	0.789 ± 0.022	0.758 ± 0.034	0.761 ± 0.045	0.519 ± 0.014
Cat + num	0.817 ± 0.001	0.855 ± 0.001	0.749 ± 0.001	**0.996** **±0.001**	0.607 ± 0.002	0.668 ± 0.002

Values are reported as mean ± standard deviation. Five runs of results are averaged. The best outcomes for each evaluation metric are in bold.

We see that training with reduced features may achieve higher metrics in some cases. Notably, training without descriptions has a higher F1-score than the original model but has a lower precision. Also, training without tweets has a higher recall value. Training without numerical properties has a higher precision and specificity but a lower MCC than training without description. Training with only the categorical and numerical properties has the highest recall. Therefore, training with combinations of features does not achieve as high metrics as training with all the features in each case, meaning that all features contribute to the model's performance. These remarks are important to consider for future research in ensuring the dataset's quality, but training the model with all the features provided makes it more adaptable to other datasets. For further understanding we will train the model using only one feature at a time, to investigate their importance separately. We present the results in Table 7.

**Table 7:** Training model with only one feature.

**Model**	**Accuracy**	**F1-score**	**Precision**	**Recall**	**Specificity**	**MCC**
Ours	**0.857** **±0.004**	**0.871** **±0.003**	**0.849** **±0.008**	0.895 ± 0.007	**0.812** **±0.013**	**0.712** **±0.007**
only description	0.699 ± 0.007	0.74 ± 0.008	0.695 ± 0.015	0.793 ± 0.033	0.589 ± 0.046	0.392 ± 0.014
only tweets	0.585 ± 0.011	0.643 ± 0.017	0.602 ± 0.008	0.691 ± 0.037	0.461 ± 0.033	0.157 ± 0.022
only numerical	0.679 ± 0.02	0.758 ± 0.013	0.641 ± 0.018	0.929 ± 0.034	0.385 ± 0.063	0.383 ± 0.039
only categorical	0.817 ± 0.001	0.853 ± 0.001	0.747 ± 0.001	**1.000** **±0.001**	0.6 ± 0.001	0.667 ± 0.001

Values are reported as mean ± standard deviation. Five runs of results are averaged. The best outcomes for each evaluation metric are in bold.

Obviously, the model trained with all the features has the best performance. From the results, we deduce that the categorical property is the feature that contributes the most to the model's sufficient accuracy. This ablation study proves that all features are advantageous for training our model to perform well in the task of bot detection. However, they do not contribute equally, and more studies to enhance the quality of the datasets could benefit future studies of bot detection.

Next, we compare the architecture that results from the architecture search with a Gate operation and without a Gate operation. The findings of this ablation study are depicted in Table 8. We see that the architecture without the gate has a reduced accuracy by 0.5% compared to the model's and a reduced F1-score by 0.46%. The gating mechanism dynamically consolidates information from all propagation steps, effectively regulating the smoothness of various nodes. Without it, the T operations take as input only the last output of the P steps. This is the reason the model underperforms without the Gate operation in the P functions, as it may suffer from over-smoothing. The architectures that are examined during this search have more T steps and shallower propagation processes, failing to obtain information from nodes during message passing as successfully as the original model. This ablation study proves the importance of the Gate operation in the P functions during our architecture search.

**Table 8:** Ablation study on gate operation.

**Model**	**Accuracy**	**F1-score**	**Precision**	**Recall**	**Specificity**	**MCC**
With gate	**0.857** **±0.004**	**0.871** **±0.003**	**0.849** **±0.008**	**0.895** **±0.007**	**0.812** **±0.013**	**0.712** **±0.007**
Without gate	0.853 ± 0.003	0.867 ± 0.004	0.845 ± 0.010	0.891 ± 0.016	0.808 ± 0.018	0.704 ± 0.007

Values are reported as mean ± standard deviation. Five runs of results are averaged. The best outcomes for each evaluation metric are in bold.

Finally, we compare the architecture that results from the architecture search with a skip-connection operation and without a skip-connection operation. The findings of this ablation study are depicted in Table 9. We see that the architecture without the gate has a reduced accuracy by 0.93% compared to the model's and a reduced F1-score by 1.2%. Without the skip-connection operation, the input of the T steps is only the output of the last step. This may lead to the degradation of the model as the transformation functions can increase. The processing of the messages from nodes is not as effective and the accuracy declines. This ablation study proves the importance of the skip-connection operation in the T functions during our architecture search.

**Table 9:** Ablation study on skip-connection operation.

**Model**	**Accuracy**	**F1-score**	**Precision**	**Recall**	**Specificity**	**MCC**
With skip	**0.857** **±0.004**	**0.871** **±0.003**	0.849 ± 0.008	**0.895** **±0.007**	0.812 ± 0.013	**0.712** **±0.007**
Without skip	0.849 ± 0.009	0.860 ± 0.01	**0.857** **±0.010**	0.863 ± 0.026	**0.831** **±0.017**	0.695 ± 0.018

Values are reported as mean ± standard deviation. Five runs of results are averaged. The best outcomes for each evaluation metric are in bold.

## 9 Discussion

### 9.1 Implications

The proliferation of social media bots has prompted concerns regarding user safety and their broader societal impact. Bot detection, a focal point of contemporary studies, is not only explored through the lens of machine learning but also delves into the realms of social science. Various methodologies have been employed, encompassing supervised or unsupervised learning or a hybrid of both. A relatively recent and innovative approach involves Graph Neural Network (GNN)-based architectures, integrating diverse user features and interactions to construct a comprehensive graph representation. In our work, we formulate a heterogeneous graph that captures the following relationships between users, incorporating nodes with information on user profiles, tweets, and interests. This novel contribution enhances existing bot detection research by demonstrating the efficacy of integrating and analyzing user relationships.

As technology advances, the adaptive nature of bots poses an ongoing challenge for detection models, rendering many state-of-the-art architectures ineffective against newer datasets. The pressing need for adaptable models underscores the importance of overcoming the limitations associated with fixed architectures. Neural Architecture Search (NAS) models prove to be a promising solution, demonstrating their potential to enhance model efficiency in real-world tasks by automatically searching through various architectures. Historically, the adoption of NAS techniques for bot detection is limited, so we propose the implementation of an adapted DFG-NAS. By integrating DFG-NAS and tailoring it to Relational Graph Convolutional Networks, we explore optimal permutations of Propagation and Transformation steps in the message-passing protocol of the RGCN layers. Our investigation showcases superior performances of the top architectures compared to state-of-the-art models. Our work is one of the starting points in implementing architecture search models on bot detection. Our research findings encourage further exploration into how NAS models can automatically construct more effective architectures, resulting in a future restraint of the existence of bots.

### 9.2 Applicability of our approach to different types of social interaction

In this section, we examine the applicability of our introduced approach to other types of social interaction besides social media.

- Online gaming: bots impersonate human players to manipulate game outcomes. Bots are capable of playing without breaks. Therefore, they are able to gather resources, items, and so on very quickly which help them go to the next stage of gaming (Kang et al., 2013). Thus, people end playing with bots; so, it is impossible to win them. This fact entails serious issues, i.e., unfair gaming. Therefore, the early detection of bots in gaming is crucial, in order to ensure fair play in competitive and multiplayer games. Our method could be adapted by using response times, movement patterns, and time-series data as input features.
- Customer reviews and rating platforms: bots are often used for creating fake reviews and inflating rating in review platforms, including Amazon and Yelp. The main aim of bots is to promote specific products, restaurants, and so on. Our approach could be easily adapted to this case, since textual, timing, and user behavior features will be used.
- Digital voting and polling systems: bots are used to alter the results of Internet Polling (Mohammadi and Abbasimehr, 2010). Therefore, early recognition of bots in voting is crucial, so as to ensure reliable outcomes. Our method can be adapted by integrating features, such as IP addresses, voting patterns, and timing.
- Email and messaging systems: bots are responsible for spam and phishing. Early detection of bots is crucial for enhancing security. Features, including email headers, IP addresses, etc., must be incorporated in our study.

### 9.3 Limitations

Our study comes with some limitations. Firstly, we conducted our experiments only on one dataset, which does not ensure generalizabilty of our proposed approach. Therefore, in the future, we aim to test our method on TwiBot-22 dataset (Feng et al., 2024). Secondly, our method is based on the collection of labeled data. Obtaining labeled data is a difficult task. For this reason, unsupervised and self-supervised learning algorithms have been developed for addressing the issue of labels' scarcity. Applying unsupervised and self-supervised learning in conjunction with our approach is one of our future plans. Thirdly, we did not tune the hyperparameters due to limited access to GPU resources. Hyperparameter tuning ensures that optimal performance is obtained. Finally, we represented each user as a concatenation of features. Concatenation does not capture the inherent correlation of the different modalities. In the future, we aim to use multimodal fusion methods for constructing each user's representation (Ilias et al., 2022; Ilias and Askounis, 2023a; Chatzianastasis et al., 2023).

## 10 Conclusions and future work

As social media continues to play a pivotal role in shaping public opinion and discourse, the development of effective and adaptive bot detection methods becomes increasingly crucial for maintaining the integrity and trustworthiness of online information. In this study, we introduced a novel model for identifying bots, integrating GNNs and NAS algorithms, demonstrating significant performance gains. The integration of Graph Neural Architecture Search empowered us to dynamically determine optimal combinations of propagation and transformation operations in the graph neural network architecture. This adaptive architecture effectively addresses the constraints imposed by fixed structures, introducing a level of flexibility essential for improving the performance on the bot detection task. From the experiment results we conclude that the five architectures with the highest validation accuracy, during the architecture search, are quite efficient in our task and compete with other models. Meanwhile, the one with the highest accuracy achieves a test accuracy of 85.68%, surpassing other state-of-the-art models for bot detection. The outcomes of the experiment present promising prospects for integrating more Neural Architecture Search (NAS) methods into the domain of bot detection in various social media platforms.

The exploration of dynamic graph adaptations stands as a crucial avenue for future research in the task of bot identification in social media platform X. The dynamic nature of social networks, characterized by the continuous incorporation of new users, necessitates the development of mechanisms to seamlessly integrate these additions into the evolving graph structure. Investigating methods for real-time graph updates and exploring how the model adapts to the inclusion of new users will enhance the system's agility in capturing emerging bot behaviors within the dynamic social landscape. Furthermore, the prospect of transferring our model to other social media platforms emerges as a key future avenue. Extending the applicability of our approach beyond X involves understanding the unique dynamics and characteristics of different platforms. Future work should focus on developing a transferable framework capable of recognizing bot-like behaviors across diverse social networks. By addressing the nuances and variations in user interactions and content features, we can contribute to the development of a versatile bot detection system with broader applications in the ever-expanding realm of social media platforms.

## Data Availability

Publicly available datasets were analysed in this study. This data can be found here: https://github.com/BunsenFeng/TwiBot-20. Please contact shangbin@cs.washington.edu to obtain permission to download the dataset for research efforts only.
